# How well do face masks worn by children block the release of exhaled particles?

**Published:** 2023-05

**MAY 3, 2023 -** New research published in Pediatric Investigation provides evidence that face masks reduce the release of exhaled particles when used by school-aged children.

For the study, 23 healthy children were asked to perform activities that ranged in intensity (breathe quietly, speak, sing, cough, and sneeze) while wearing no mask, a cloth mask, or a surgical mask.

The production of exhaled particles that were 5 µm or smaller, which is the dominant mode of transmission of many respiratory viruses, increased with coughing and sneezing. Face masks—especially surgical face masks—effectively reduced the release of these and other sized particles.

“Understanding the factors that affect respiratory particle emission can guide public health measures to prevent the spread of respiratory infections, which are a leading cause of death and hospitalization among young children worldwide,” said corresponding author Peter P. Moschovis, MD, MPH, of Massachusetts General Hospital and Harvard Medical School.

***Link to Study: https://onlinelibrary.wiley.com/doi/10.1002/ped4.12376***

***Full citation:** “The effect of activity and face masks on exhaled particles in children.” Peter P. Moschovis, Jesiel Lombay, Jennifer Rooney, Sara R. Schenkel, Dilpreet Singh, Shawheen J. Rezaei, Nora Salo, Amanda Gong, Lael M. Yonker, Jhill Shah, Douglas Hayden, Patricia L. Hibberd, Philip Demokritou, T. Bernard Kinane. Pediatr Investig; Published Online: MAY 3, 2022 (DOI: 10.1002/ped4.12376).*

*Copyright © 2019 The Cochrane Collaboration. Published by John Wiley & Sons, Ltd., reproduced with permission.*

